# Effect of different brewing times on antioxidant activity and polyphenol content of loosely packed and bagged black teas (*Camellia sinensis *L.)

**Published:** 2016

**Authors:** Zeinab Nikniaz, Reza Mahdavi, Seyed Jamal Ghaemmaghami, Neda Lotfi Yagin, Leila Nikniaz

**Affiliations:** 1 *Liver and Gastrointestinal disease research center, Tabriz University of medical sciences, Tabriz-Iran*; 2 *Nutrition Research Centre, Tabriz University of Medical Sciences, Tabriz-Iran*; 3 *Nutrition Research Centre, Tabriz University of medical sciences, Tabriz-Iran*; 4 *Student Research Committee, Tabriz University of medical sciences, Tabriz-Iran*; 5 *Tabriz Health Services Management Research Center, Tabriz University of Medical Sciences, Tabriz-Iran*

**Keywords:** *Antioxidant*, *Polyphenol*, *Tea*, *Infusion time*

## Abstract

**Objective:**

Determination and comparison of the effect of infusion time on the antioxidant activity and total polyphenol contents of bagged and loosely packed black teas.

**Materials and Methods:**

For twenty loosely packed and eleven bagged tea samples, the antioxidant activity and total polyphenol content were analyzed using FRAP and Folin-Ciocalteau methods, respectively. The ANOVA with Tukey post-hoc test and independent t-test were used for statistical analysis.

**Results:**

The antioxidant activity and polyphenol content of various brands of tea samples were significantly different. There were significant differences in the antioxidant activity of loosely packed teas between 5, 15(p=0.03), 30(p=0.02) and 60(p=0.007) minutes of brewing times. Besides, there was a significant difference in antioxidant activity of bagged samples infused for 1 minute with four other infusion time points (p<0.001). In the case of polyphenol content, in loosely-packed tea samples, there were not significant differences between different brewing times (p=0.15). However, in bagged samples, the polyphenol contents of samples that were brewed for 1 minute were significantly lower than samples brewed for 3, 4, and 5 minutes (p<0.05). The antioxidant activity and polyphenol content of tea bags were significantly higher than those ofloosely-packed forms of the same brands at 5-min of brewing time (p<0.001).

**Conclusion:**

The infusion time and the form of tea (loosely packed or bagged) were shown to be important determinants of the antioxidant activity and polyphenol content of black tea infusions in addition to the variety, growing environment and manufacturing conditions.

## Introduction

Tea (*Camellia sinensis L.*), mainly black tea, is the second most popular beverage throughout the world after water due to its refreshing and mild stimulant effects (Muktar and Ahmad, 2000). Since it contains bioactive compounds with health promoting effects, tea has anti-oxidative, anti-carcinogenic, anti-hypertensive, anti-mutagenic, and angiogenesis inhibitor activities (Halder et al., 2005; Han, 1997; Sarkar and Bhaduri, 2001; Chung et al., 1998, Sakanaka et al., 2000). The beneficial health effects of tea are ascribed mainly to its antioxidant properties (Yashin, 2011). Numerous studies have shown that the antioxidant activity of tea may be due to its polyphenols (Nihal et al., 2005; Karakaya and Kavas, 1999). 

Polyphenols are important natural antioxidants. They represent a group of secondary metabolites widely distributed in the medicinal plants, vegetables, fruits and a variety of beverages such as fruit juices, wine and tea (Scalbert et al., 2005; Mahdavi et al., 2011, Tsao and Yang, 2003, Benzie and Strain, 1996; Singleton et al., 1965). The antioxidant activity of dietary polyphenols is considered to be much greater than that of the vitamins (Tsao and Yang, 2003). 

The growing interest in the potential health benefits of tea together with its popularity as a beverage have driven numerous investigations to discover the constituents of tea and their biological properties (Gupta et al., 2002). There have been some reports on the antioxidant activity and polyphenol content of black tea (Yashin et al., 2011; Khokhar and Magnusdottir, 2002; Chan et al., 2007). However, little or no information is available on the effect of infusion time on these properties. It seems that brewing time and techniques, which likely differ greatly from region to region, can affect the antioxidant activity and polyphenol content of the final product. Studying the influence of brewing time on antioxidants activity and polyphenol contents of tea can lead to more information on how to prepare the product more effectively. Besides, although many tea drinkers continue to prepare their drink using loose-leaf tea, the more popular way of making tea is by using teabags. So, this study was primarily conducted to determine and compare the effect of infusion time on the antioxidants activity and total polyphenol contents of bagged and loosely packed black tea samples. 

## Materials and Methods

**Samples**

Thirty one commercial samples, consisting of loosely packed form of twenty different brands (A-T) and tea bags of eleven different brands (A-K) were purchased from the supermarkets in Tabriz, Iran.

**Chemicals and reagents**

All reagents and solvents used were of analytical grade and were used without further purification. Doubly distilled water was used throughout the experiment. Gallic acid was obtained from Sigma. All other materials were purchased from Merck.

Each tea bag sample was infused in 240 ml of hot tap water (80°C) for 1, 2,3, 4 and 5 minutes, and stirred twice. After infusions, the tea bag was removed. The loosely packed tea samples (2 grams), were brewed in 240 ml of 80^°^C water for 5, 10, 15, 30 and 60 minutes (which are popular brewing times in Iran). The solutions were allowed to reach the room temperature prior to analysis

**Determination of ferric reducing/antioxidant power (FRAP assay)**

The ferric reducing/antioxidant power (FRAP) assay was carried out according to Benzie and Strain (1996) (Iris et al., 1996). The FRAP assay is based on the reduction of the Fe^3+^-2,4,6-tripyridyl-S-triazine complex to the ferrous form (Fe^2+^) and the intensity of the reaction is monitored by measuring the change in absorption at 593 nm. A FRAP reagent was prepared by mixing acetic buffer, TPTZ and FeCl_3_.6H_2_O (20 mM water solution) at a ratio of 10:1:1. Brieﬂy, to a volume of 30 µl of tea extract, 1 ml of FRAP reagent was added. After 4 min, the absorbance of blue coloration was measured against a blank sample. A standard curve was prepared using different concentrations (0.1–1 mM) of Fe^2+^. All measurements were performed in triplicate.

**Determination of total polyphenol content**

The total polyphenol contents of tea extract were determined by using Folin-Ciocalteu method (Singleton et al., 1965). Briefly, 1 ml of extract or standard solution of gallic acid (20, 40, 60, 80 and 100 mg/l) was decanted in 25 ml volumetric flask containing 9 ml of distilled deionized water. Then, 1 ml of Folin-Ciocalteu reagent was added to the mixture and the mixture was shaken. After 5 min, 10 ml of 7% Na_2_CO_2_ solution was added and the solution was diluted to the volume with distilled deionized H_2_O and mixed. After incubation for 90 min at room temperature, the absorbance against prepared reagent blank (distilled deionized H_2_O) was measured at 750 nm. All measurements were performed in triplicate.

**Statistical analysis**

Data were expressed as means±standard error of the mean of three independent experiments carried out in triplicate. The one-way ANOVA with Tukey post-hoc test was used to compare the total polyphenol and antioxidant contents of different tea brands and also between different infusion times. The independent t- test was used to compare the antioxidant activity and polyphenol contents between black loosely packed and tea bags. P-values of less than 0.05 were considered statistically significant.

## Results

The obtained data for antioxidant activity of black teas (loosely packed and bagged forms) are presented in Table 1. In the case of loosely packed black teas, the highest antioxidant activity was found in the samples from brand N (863.44±0.02 mM Fe(II)/(2g/240ml)) at 60 minutes of brewing time and in the case of bagged forms, in the samples from brand E (857.61±0.01 mM Fe(II)/(2g/240ml)) at 5 minutes of brewing time. According to the results of one-way ANOVA and post-hoc Tukey analysis, there were significant differences in antioxidant activities among different brands of loosely packed and bagged black teas (p<0.001). 

**Figure 1:**
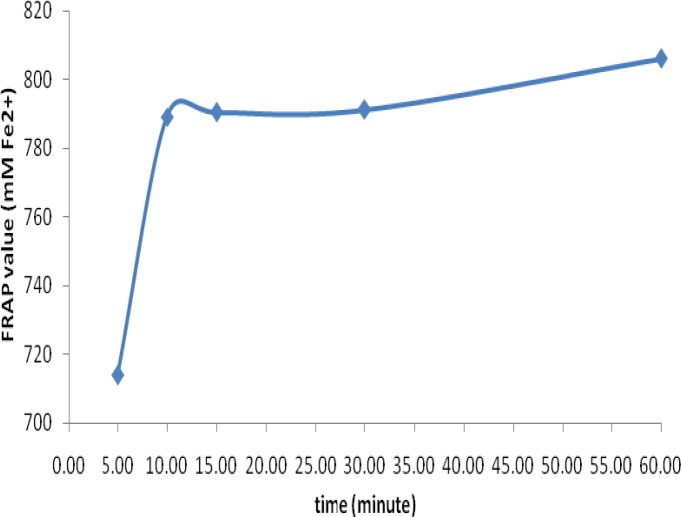
The effect of different infusion time on the mean antioxidant activity (mMFe(II)/(2g/240ml)) of loosely packed black tea

As depicted in Figure 1, the mean antioxidant activity of loosely packed black tea extracts significantly depends on the time of extraction. The mean antioxidant activity of loosely packed black teas at 5, 10, 15, 30 and 60 minutes of infusion times were 713.95±20.26, 789.13±8.28, 790.39±11.28, 791.17±13.95 and 806.06±8.57 mM Fe(II)/(2g/240ml), respectively. The most intensive extraction of antioxidants of loosely packed black tea took place in the first 10 minutes and then, the rate of extraction diminished. The results of one-way ANOVA analysis showed that there were statistically significant differences in the antioxidant activity among different brewing times (p<0.006) and post-hoc analysis further specified that there was significant difference in the antioxidant activity of tea between 5 minutes of brewing time and 15 (p=0.03), 30 (p=0.02) and 60 (p=0.007) minutes of brewing times. 

**Table1:** Antioxidant activity (mMFe(II)/(2g/240ml)) of loosely packed and bagged black tea after different brewing times 2g/240ml

**Tea brand**	**Infusion time**					
	**1 minute**	**2minutes**	**3 minutes**	**4 minutes**	**5 minutes**	**p-value** *
**Tea bags**						
**A**	653.96±0.01	694.56±0.01	713.03±0.01	714.87±0.01	717.74±0.01	<0.001
**B**	718.86±0.01	776.65±0.02	794.90±0.02	799.58±0.01	812.72±0.02	<0.001
**C**	638.99±0.02	741.60±0.01	761.74±0.01	777.42±0.02	791.15±0.01	<0.001
**D**	757.92±0.01	810.97±0.01	831.96±0.01	834.29±0.02	854.70±0.01	<0.001
**E**	778.32±0.02	842.45±0.02	845.37±0.01	843.62±0.01	857.61±0.01	<0.001
**F**	688.54±0.01	812.72±0.01	822.20±0.02	825.63±0.01	842.55±0.02	<0.001
**G**	706.62±0.01	717.69±0.01	741.60±0.01	773.08±0.02	788.82±0.01	<0.001
**H**	774.24±0.02	775.99±0.02	783.57±0.01	786.26±0.01	791.18±0.01	<0.001
**I**	677.47±0.01	699.04±0.02	798.73±0.02	844.20±0.02	910.67±0.02	<0.001
**J**	606.31±0.01	680.96±0.01	689.71±0.01	733.57±0.01	789.26±0.01	<0.001
**K**	658.81±0.02	775.99±0.01	793.53±0.01	793.84±0.01	850.34±0.01	<0.001
**p-value** *	<0.001	<0.001	<0.001	<0.001	<0.001	
	Infusion time					P-VALUE*
**Loose tea**	5 minutes	10 minutes	15 minutes	30 minuets	60 minutes	<0.001
**A**	616.25±0.02	686.79±0.01	690.35±0.01	693.78±0.02	738.68±0.01	<0.001
**B**	745.09±0.01	784.15±0.02	796.98±0.02	794.65±0.01	823.22±0.02	<0.001
**C**	779.49±0.01	812.14±0.01	838.40±0.01	882.99±0.01	898.73±0.01	<0.001
**D**	791.73±0.01	809.81±0.02	827.88±0.02	835.46±0.02	842.76±0.02	<0.001
**E**	437.27±0.02	714.47±0.01	781.74±0.02	784.82±0.02	785.41±0.01	<0.001
**F**	529.97±0.01	829.05±0.02	842.45±0.01	845.95±0.01	858.20±0.02	<0.001
**G**	699.04±0.01	780.07±0.02	816.80±0.01	823.80±0.02	829.05±0.01	<0.001
**H**	746.84±0.02	818.55±0.01	831.96±0.01	851.78±0.01	854.47±0.01	<0.001
**I**	700.20±0.02	765.50±0.01	771.33±0.02	779.49±0.02	795.81±0.01	<0.001
**J**	701.37±0.01	717.69±0.02	753.26±0.01	753.43±0.02	757.51±0.02	<0.001
**K**	678.63±0.01	739.26±0.01	755.59±0.02	753.84±0.01	755.59±0.01	<0.001
**L**	819.13±0.01	837.21±0.01	850.62±0.02	852.45±0.01	857.12±0.02	<0.001
**M**	812.14±0.02	826.71±0.01	838.96±0.01	854.70±0.02	860.21±0.02	<0.001
**N**	714.19±0.01	799.90±0.02	825.55±0.01	835.64±0.02	863.44±0.02	<0.001
**O**	796.98±0.02	808.64±0.01	822.63±0.02	822.63±0.01	851.20±0.01	<0.001
**P**	732.54±0.01	787.65±0.01	806.89±0.02	823.75±0.02	841.87±0.02	<0.001
**Q**	741.60±0.01	791.73±0.02	795.81±0.01	796.35±0.01	799.90±0.01	<0.001
**R**	765.50±0.02	803.98±0.02	805.14±0.02	832.54±0.02	838.46±0.02	<0.001
**S**	766.66±0.01	829.05±0.02	851.51±0.01	854.38±0.01	856.22±0.01	<0.001
**T**	762.00±0.02	790.57±0.01	797.74±0.02	797.83±0.01	806.58±0.02	<0.001
**p-value** *	<0.001	<0.001	<0.001	<0.001	<0.001	

All analytical data are the mean of triplicate measurements of three independent samples ±SD.

* p-value of one-way ANOVA for different brewing times.

** p-value of one-way ANOVA for different brands.

**Figure 2:**
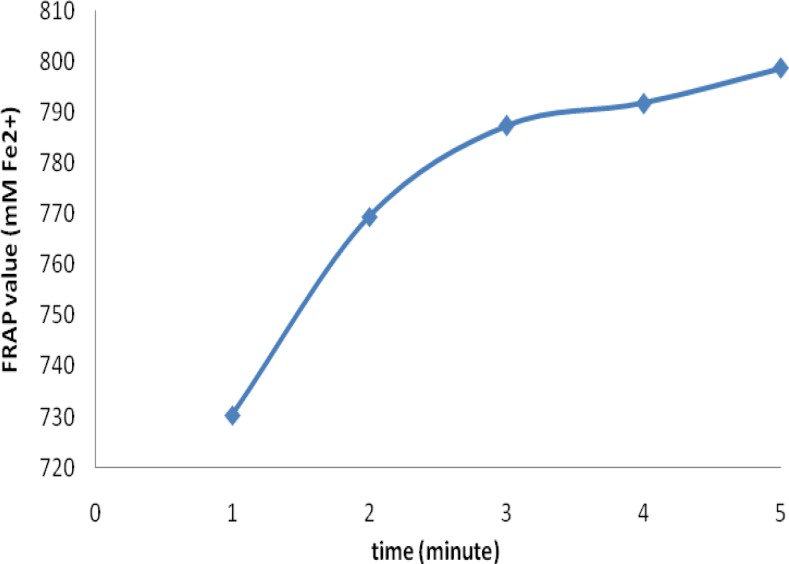
The effect of different infusion times on the mean antioxidant activity (mMFe(II)/(2g/240ml)) of bagged black tea

In the case of bagged teas, as shown in Figure 2, the mean antioxidant activity were 730.37±13.20, 769.40±15.23, 787.39±12.48, 791.73±11.94 and 798.70±16.16 mM Fe(II)/(2g/240ml) at 1, 2, 3, 4 and 5 minutes of infusion times, respectively. The highest antioxidant activity was at first 3 minutes of brewing time. The results of one-way ANOVA analysis showed that there were statistically significant differences in the antioxidant activity among different brewing times (p<0.001) and post-hoc analysis further indicated that there was a significant difference in antioxidant activity of samples infused for 1 minutes and the other four infusion time points (p<0.001).

The total phenolic contents of different loosely packed and bagged black tea samples were shown in Table 2. In the loosely packed tea samples, brand N (40.78±0.01 mg GAE/240mL) at the 60 minutes of brewing time and in the bagged form, at 5 minutes of infusion time, brand G (71.92±0.01 mg GAE/240mL) had the highest amount of polyphenol contents. Statistical analysis indicated that there was a significant difference in the polyphenol content among different brands of both loosely packed and bagged black tea samples (p<0.001). 

**Table 2:** Polyphenol (mg GAE/240mL) content of loosely packed and bagged black tea after different brewing times

**Tea brand**		**Infusion time**									
**Tea bags**		**1 min**	**2 min**		**3min**		**4min**		**5min**		**p-value** *
**A**		23.98±0.01	23.64±0.01		27.06±0.01		36.40±0.01		71.92±0.01		>0.05
**B**		27.16±0.02	36.95±0.02		38.74±0.01		42.53±0.01		66.34±0.02		>0.05
**C**		13.30±0.01	18.890.02		19.26±0.01		26.07±0.02		31.10±0.02		>0.05
**D**		16.36±0.01	18.74±0.02		19.35±0.02		21.29±0.02		29.52±0.01		>0.05
**E**		17.53±0.01	17.90±0.01		18.34±0.02		21.97±0.01		27.31±0.02		>0.05
**F**		21.78±0.02	23.64±0.02		27.06±0.01		29.40±0.02		71.92±0.01		>0.05
**G**		23.98±0.02	24.64±0.01		27.91±0.01		31.45±0.02		69.73±0.01		>0.05
**H**		17.16±0.01	21.940.02		21.72±0.02		26.53±0.01		66.41±0.02		>0.05
**I**		13.30±0.01	18.89±0.01		19.26±0.02		26.07±0.02		31.10±0.01		>0.05
**J**		6.36±0.02	8.743±0.01		9.35±0.02		11.29±0.02		13.52±0.01		>0.05
**K**		17.53±0.01	17.90±0.02		18.34±0.01		21.97±0.01		27.34±0.02		>0.05
**p-value** *		<0.001	<0.001		<0.001		<0.001		<0.001		
**Infusion time**											
**Loose tea**	5 minutes			10 minutes		15 minutes		30 minutes		60 minutes	p-value*
**A**	18.90±0.01			23.72±0.02		28.31±0.01		28.84±0.01		30.65±0.01	<0.001
**B**	19.21±0.01			27.31±0.01		27.76±0.01		33.84±0.01		34.69±0.02	<0.001
**C**	13.25±0.02			27.10±0.02		28.10±0.02		30.26±0.02		31.83±0.01	<0.001
**D**	18.39±0.01			21.70±0.02		24.60±0.01		25.92±0.02		33.59±0.02	<0.001
**E**	21.35±0.01			29.24±0.01		30.25±0.02		30.63±0.02		40.35±0.01	<0.001
**F**	17.90±0.01			22.71±0.02		27.80±0.01		29.16±0.01		40.68±0.02	<0.001
**G**	21.23±0.01			29.16±0.02		33.02±0.01		35.26±0.02		38.36±0.01	<0.001
**H**	18.54±0.01			22.46±0.02		23.65±0.01		25.13±0.01		34.08±0.01	<0.001
**I**	8.90±0.02			13.75±0.01		18.31±0.02		18.84±0.02		19.65±0.02	<0.001
**J**	19.21±0.02			27.3±0.011		27.76±0.02		33.84±0.02		34.69±0.02	<0.001
**K**	13.22±0.02			18.10±0.02		27.10±0.02		30.26±0.01		31.83±0.01	<0.001
**L**	18.39±0.02			20.70±0.01		21.60±0.02		24.92±0.01		33.596±0.02	<0.001
**M**	21.35±0.02			25.24±0.02		29.25±0.01		30.63±0.01		40.33±0.01	<0.001
**N**	17.90±0.01			22.71±0.02		27.80±0.01		29.16±0.02		40.78±0.01	<0.001
**O**	21.23±0.02			29.16±0.01		33.04±0.01		34.26±0.02		38.36±0.01	<0.001
**P**	17.42±0.02			22.46±0.02		23.65±0.02		24.13±0.02		34.08±0.02	<0.001
**Q**	15.02±0.01			17.69±0.02		21.72±0.02		26.32±0.01		27.80±0.02	<0.001
**R**	16.91±0.02			18.60±0.02		23.72±0.02		25.79±0.01		30.39±0.01	<0.001
**S**	16.54±0.02			24.47±0.02		24.92±0.01		29.48±0.02		30.22±0.01	<0.001
**T**	23.73±0.01			23.03±0.01		25.87±0.01		30.63±0.02		30.61±0.01	<0.001
**p-value** *	<0.001			<0.001		<0.001		<0.001		<0.001	

All analytical data are the mean of triplicate measurements of three independent samples ±SD.

* p-value of one-way ANOVA for different brewing times.

** p-value of one-way ANOVA for different brands.

**Figure 3:**
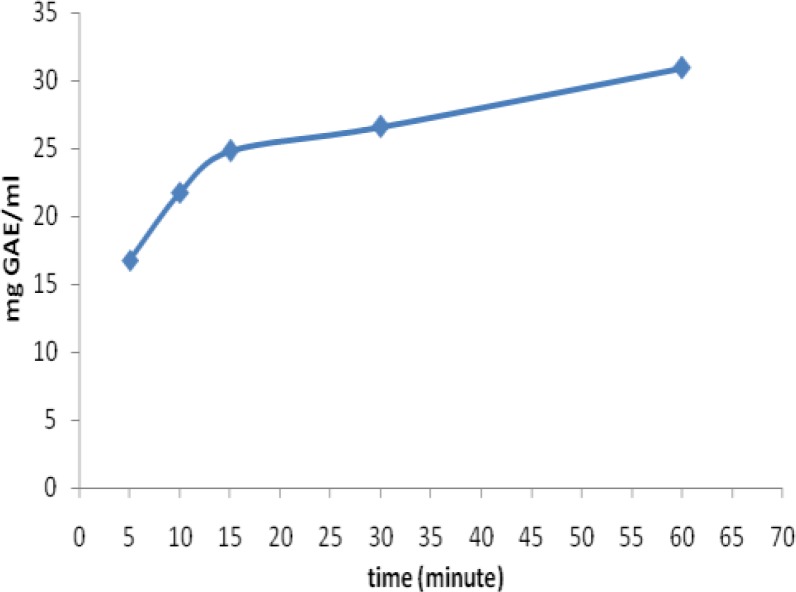
The effect of different infusion times on the mean polyphenol content (mg GAE/(2g/240ml)) of loosely packed black tea

**Figure 4:**
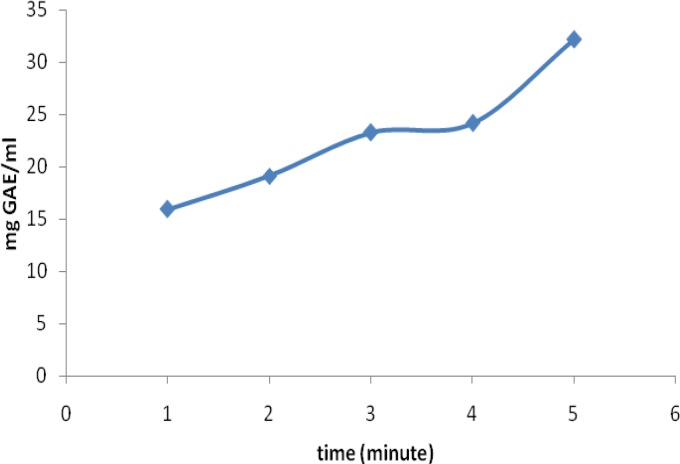
The effect of different infusion times on the mean polyphenol (mg GAE/(2g/240ml)) content of bagged black tea

The mean polyphenol contents of loosely packed and bagged black teas at different brewing times were shown in Figures3 and 4, respectively. As presented in these figures, the mean polyphenol content of black tea samples significantly depends on the time of extraction. In the loosely packed black teas at 5, 10, 15, 30 and 60 minutes of infusion time, the mean polyphenol contents were 16.77±1.27, 21.78±1.57, 24.80±1.61, 26.62±1.54 and 30.95±2.12 mg GAE/240ml, respectively. The highest level of polyphenol content was extracted from the loosely packed tea in the first 15 minutes. Based on the results of one-way ANOVA, there were not statistically significant differences in the polyphenol content among different brewing times in loosely packed black tea samples (p=0.15).

In the case of bagged samples, the mean polyphenol contents were 15.91±2.40, 19.10±2.51, 23.36±3.78, 24.14±3.01 and 32.13±8.26 mg GAE/240ml at 1, 2, 3, 4 and 5 minutes of infusion times, respectively. The highest level of polyphenol content was extracted from the bagged tea in the first 3 minutes of infusion time. The results of one-way ANOVA and post-hoc analysis revealed that the polyphenol contents of tea samples brewed for 1 minute were significantly lower than samples brewed for 3, 4, and 5 minutes (p<0.05). Besides, there was a significant difference between samples brewed for 2 minutes and samples brewed for 5 minutes (p=0.002). 

Comparison of the antioxidant activity and polyphenol content of five same brands of loosely packed black teas and tea bags are shown in Table 3. 

**Table 3:** comparison of the antioxidant activity (mM Fe(II)/(2g/240ml)) and polyphenol content (mg GAE/240mL) of the same brands of loosely packed and bagged black tea at 5 minutes of infusion

**Different brands**		**Loose tea**	**Bagged tea** ** (the values corrected to 2 g of tea)**	p-value*
1	Antioxidant activity	779.49±0.01	798.18±0.01	<0.001
	Polyphenol content	13.251±0.02	65.22±0.02	<0.001
2	Antioxidant activity	766.66±0.01	833.14±0.01	<0.001
	Polyphenol content	16.54±0.02	21.16±0.01	<0.001
3	Antioxidant activity	732.54±0.01	811.67±0.02	<0.001
	Polyphenol content	17.42±0.02	65.23±0.02	<0.001
4	Antioxidant activity	819.13±0.01	909.35±0.02	<0.001
	Polyphenol content	18.396±0.02	30.43±0.01	<0.001
5	Antioxidant activity	766.66±0.01	849.14±0.01	<0.001
	Polyphenol content	16.546±0.02	25.54±0.01	<0.001

p-value of independent t-test of the same brands of loosely packed teas and tea bags corrected to 2 gram of tea.

The independent t-test showed that the antioxidant activity and polyphenol content of tea bags were significantly higher than thoseof the same brands of loosely packed forms after5minutes of brewing (p<0.001).

## Discussion

Because of the preventive potential of the antioxidant products against a large number of diseases; the total content of antioxidants in foods, beverages, and herbal extracts has been widely studied (Yashin et al., 2011). In this regard, there are many reports describing the antioxidant activity and polyphenol contents of black teas (Yashin et al., 2011; Khokhar and Magnusdottir , 2002; Chan et al., 2007) and it was shown that black tea contains high amount of antioxidant and polyphenol compounds. In the present study, it was also confirmed that bagged and loosely packed black tea are good sources of these components. However, there were significant differences in the antioxidant activity and polyphenol content of different brands of loosely packed and bagged black tea samples. These findings are inline with other studies conducted on different brands of loosely packed and bagged teas in Malaysia (Chan et al., 2007), UK (Ryan et al., 2010) and Argentina (Anesini et al., 2008). These differences among brands may be due to differences in the plant variety, growth conditions, and processing methods which would be expected to produce quite wide variations in the chemical compositions of the resulting products. Besides, different methods of manufacturing also account for the marked difference in the chemical compositions of black teas (Cloughley, 1981; Astill et al., 2001; Yao et al., 2006; Cheong et al., 2005; Lachman et al., 2003; Armoskaite et al., 2011; Kyle et al., 2007). In the case of tea bags, the quality of the paper used for packaging teabags is also important (Yao et al., 2006). 

The results of the present study showed that, although the product itself can have a significant effect, the preparation variables also greatly influence the antioxidant activity and polyphenol content of the infusion. Previously published reports on green teas showed that the antioxidant capacity and the total polyphenols content in tea extracts correlate with extraction time (Cheong et al., 2005, Lachman et al., 2003, Armoskaite et al., 2011). However, there are limited data about the effects of different infusion times on extraction of bioactive compounds from black tea (Kyle et al., 2007). As well, while previous investigations were focused on shorter times of extraction (up to 30 minutes), in present study the longer extraction times (up to 60 minutes) were also investigated to simulate extraction conditions usually used for extraction of black tea at home. Our results showed that at the first time point, more than 40% of water-soluble phenolics were extracted from both bagged and loosely packed tea samples. About 10 minutes of infusion time for loosely packed teas and 2 minutes for bagged forms were enough for the extraction of almost more than half of water-soluble phenolics under the test conditions. The observed effects of increasing phenols and antioxidant levels with increasing infusion times were also known to be influenced by stirring duration and intensity, leaf size, and tea bag porosity (Astill et al., 2001; Hakim et al., 2000). 

There appears to be no published study on comparing the antioxidant activity and polyphenol content of different forms (loosely packed and bagged) of black tea. In the previous studies on green teas, the extraction efficiency of antioxidants was significantly affected by the form (bagged or loosely packed) of tea. However, the results were controversial. For example, Rusak et al. (2008) showed that the extraction of catechins (type of polyphenols) and methylxantines from loosely packed green teas was more efficient than that of bagged forms (Rusak et al., 2008). This may be due to direct transportation of soluble components from tea leaves into the water in loosely packed teas and the flow resistance caused by the packed bed of leaves and the bag material of tea bags (Astill et al., 2001). In contrast, Rusak et al. (2008) showed that bagged green tea was a richer source of phenolic compounds and has higher antioxidant activity than loosely packed ones. Similar to the last finding, in the present study, we also showed that the extraction of antioxidant and polyphenol components from bagged black tea was more effective than that from loosely packed form. Considering the extraction dynamics of different tea forms, as tea of small particle size has been usually used in manufacturing bagged tea, this observation may be due to larger specific area of small particle tea, thus allowing a faster extraction efficacy of various tea constituents. Besides, the teabag design is likely to influence the composition of the tea infusion (Astill et al., 2001). In this regard, over the past decades, tea bag manufacturers have made a number of changes is shapes, sizes, and materials of the teabag itself for increasing the extraction efficacy of tea constituents.

## References

[1] Anesini C, Ferraro GE, Filip R (2008). Total Polyphenol Content and Antioxidant Capacity of Commercially Available Tea (Camellia sinensis) in Argentina. J Agr Food Chem.

[2] Armoskaite V, Ramanauskiene K, Maruska A, Razukas A, Dagilyte A, Baranauskas A, Briedis V (2011). The analysis of quality and antioxidant activity of green tea extracts. J Med Plants Res.

[3] Astill C, Birch MR, Dacombe C, Humphrey PG, Martin PT (2001). Factors Affecting the Caffeine and Polyphenol Contents of Black and Green Tea Infusions. J Agr Food Chem.

[4] Benzie IFF, Strain JJ (1996). The Ferric Reducing Ability of Plasma (FRAP) as a measure of ‘‘Antioxidant Power’’: The FRAP Assay. Anal Biochem.

[5] Chan EWC, Lim YY, Chew YL (2007). Antioxidant activity of Camellia sinensis leaves and tea from a lowland plantation in Malaysia. Food Chem.

[6] Chung KT, Lu Z, Chou MW (1998). Mechanism of inhibition of tannic acid and related compounds on the growth of intestinal bacteria. Food Chem Toxicol.

[7] Cloughley JB (1981). Storage deterioration in Central African tea: the effect of some production variables on the a ﬂavin degradation. J Sci Food Agric.

[8] Cheong WJ, Park MH, Kang GW, Ko JH, Seo YJ (2005). Determination of catechin compounds in Korean green tea Infusions under various extraction conditions by high performance liquid chromatography. Bull Korean Chem.

[9] Gupta S, Saha B, Giri AK (2002). Comparative antimutagenic and anticlastogenic effects of green tea and black tea: a review. Mutat Res.

[10] Halder B, Pramanick S, Mukhopadhyoy S, Giri AK (1997). Inhibition of benzo[a]pyrene induced mutagenicity and genotoxicity multiple test systems. Food Chem Toxicol.

[11] Han C (1997). Screening of anticarcinogenic ingredients in tea polyphenols. Cancer Lett.

[12] Hakim IA, Weisgerber UM, Harris RB, Balentine D, van Mierlo CAJ, Paetau-Robinson I (2000). Preparation, composition and consumption patterns of tea-based beverages in Arizona. Nutr Res.

[13] Karakaya S, Kavas A (1999). Antimutagenic activities of some foods. J Sci Food Agri.

[14] Khokhar S, Magnusdottir SGM (2002). Total Phenol, Catechin, and Caffeine Contents of Teas Commonly Consumed in the United Kingdom. J Agri Food Chem.

[15] Kyle JA, Morrice PC, McNeill G, Duthie GG (2007). Effects of infusion time and addition of milk on content and absorption of polyphenol from black tea. J Agri Food Chem.

[16] Lachman J, Hosnedl V, Pivec V, Orsak M (2003). Polyphenol content in green, black and oolong tea (Camellia sinensis/L/kuntze) infusions in different times of tea maceration. Scientia Agriculturae Bohemica.

[17] Mahdavi R, Nikniaz Z, Rafraf M, Jouyban A (2011). Determination and comparison of the total polyphenol contents of fresh and commercial fruit juices. BFJ.

[18] Muktar H, Ahmad N (2000). Tea polyphenols: prevention of cancer and optimizing health. Am J Clin Nutr.

[19] Nihal M, Ahmad N, Mukhtar H, Wood GS (2005). Antiproliferative and proapoptotic eﬀects of ( )-epigallocatechin-3-gallate on human melanoma: Possible implications for the chemoprevention of melanoma. Int J Cancer.

[20] Rusak G, Komes D, Likic S, Horz ˇic D, Kovac M (2008). Phenolic content and antioxidative capacity of green and white tea extracts depending on extraction conditions and the solvent used. Food Chem.

[21] Ryan L, PetitLisa Ryan S, Petit S (2010). Addition of whole, semiskimmed, and skimmed bovine milk reduces the total antioxidant capacity of black tea. Nutr Res.

[22] Sakanaka S, Juneja LR, Taniguchi M (2000). Antimicrobial effects of green tea polyphenols on thermophilic spore-forming bacteria. J Bioscibioeng.

[23] Sarkar A, Bhaduri A (2001). Black tea is a powerful chemopreventor of reactive oxygen and nitrogen species: comparison with its individual catechin constituents and green tea. Bio chem. Bio phy Res.

[24] Scalbert A, Johnson IT, Saltmarsh M (2005). Polyphenols: Antioxidants and beyond. Am J ClinNutr.

[25] Singleton VL, Joseph A, Rossi, JRJA (1965). Colorimetry of total phenolics with phosphomolybdic-phosphotungstic acid reagents. Am J Enol Vitic.

[26] Singleton VL, Orthofer R, Lamuela-Raventos RM (1999). Analysis of total phenols and other oxidation substrates and antioxidants by means of Folin Ciocalteu reagent. Methods Enzymol.

[27] Tsao R, Yang R (2003). Optimization of a new mobile phase to know the complex and real polyphenolic composition: towards a total phenolic index using high-performance liquid chromatography. J chromatgr.

[28] Yashin A, Yashin Y, Nemzer B (2011). Determination of Antioxidant Activity in Tea Extracts, and Their Total Antioxidant Content. Am J Biomed Sci.

[29] Yao L, Liu X, Jiang Y, Caﬃn N, Arcy BD, Singanusong R, Datta N, Xu Y (2006). Compositional analysis of teas from Australian supermarkets. Food Chem.

